# Home-based pulmonary rehabilitation program: Effect on exercise tolerance and quality of life in chronic obstructive pulmonary disease patients

**DOI:** 10.4103/1817-1737.58955

**Published:** 2010

**Authors:** Maha Ghanem, Enace Abd ELaal, Mogedda Mehany, Kawthar Tolba

**Affiliations:** *Chest Department, Assiut University Hospital, Assiut, Egypt*; 1 *Nursing School, Assiut, Egypt*; 2 *Medical Surgical Nursing Department, Faculty of Nursing, Assiut University, 71111, Assiut, Egypt*; 3 *Adult Nursing, Faculty of Nursing, Alexandria University, Alexandria, Egypt*

**Keywords:** Chronic respiratory disease questionnaire - self - administered standardized format, pulmonary rehabilitation, quality of life

## Abstract

**BACKGROUND:**

A key component in the management of chronic obstructive pulmonary disease (COPD) patients is pulmonary rehabilitation (PR), the corner stone of which is exercise training.

**AIM:**

This study aims to evaluate the effect of a two-months, home-based PR program with outpatient supervision every two weeks, on exercise tolerance and health-related quality of life (HRQL) using Arabic-translated standardized generic and specific questionnaires in COPD patients recently recovered from acute exacerbation,

**DESIGN:**

Randomized clinical trial.

**SETTING AND SUBJECTS:**

A total of 39 COPD patients who recovered from acute exacerbation were randomly allocated either a two-month home-based PR program in addition to standard medical therapy or standard medical therapy alone in the period between July 2008 and March 2009.

**METHODS:**

Pulmonary function tests (PFTs), six-minute walk distance (6-MWD) test, Arabic-translated chronic respiratory disease questionnaire-self administered standardized format (CRQ-SAS) and quality of life scale Short Form (SF-36) were compared between 25 patients with moderate to severe COPD who underwent a two-month PR program (group 1) and 14 COPD patients who did not (group 2).

**RESULTS:**

Group 1 showed significant improvement in the 6-MWD, and HRQL scores at two months compared with the usual care patients in group 2 (*P* less than 0.05). Improvement in both CRQ-SAS and SF-36 scores were statistically significant and comparable in group 1.

**CONCLUSION:**

The supervised, post discharge, two-month home-based PR program is an effective non pharmacological intervention in the management of stable patients with COPD. The 6-MWD is a simple, inexpensive and safe test to assess physical and functional capabilities among COPD patients. HRQL can be measured in patients with COPD either by disease-specific tools that have been specifically designed for use in patients with respiratory system disorders or by generic HRQL tools that can be used across populations with a variety of medical conditions. The Arabic-translated CRQ-SAS is a new tool for assessment of Arabic-speaking patients with chronic respiratory diseases.

Chronic obstructive pulmonary disease (COPD) is characterized by airflow limitation leading to reduced ventilatory capacity and is associated with shortness of breath. Patients with severe airflow limitation and those who experience repeated acute exacerbations usually suffer from impaired quality of life, reduced exercise capacity, and increased risk of re-admission. Interventions designed to hasten recovery and improve symptoms after admission to hospital may lead to reduced use of healthcare in future and real improvement in quality of life and functional ability in breathless and vulnerable patients with COPD.[1,2]

Several publications have reviewed results of PR investigation and concluded that there is substantial evidence that PR improves exercise capacity and shortness of breath.[3]

In patients with COPD, HRQL may be particularly valuable in PR assessment. Few studies have compared the effectiveness of rehabilitation programs with accompanying lectures and different teaching methods for patients with pulmonary disease.[4] In these studies, the effects of PR on HRQL have used disease-specific questionnaires designed for patients with COPD.[5,6] These tools make it difficult to compare outcomes in studies of patients with COPD to those with other nonpulmonary disorders and some require administration by a trained interviewer. Also, the effects of early PR of outpatients in the acute recovery phase after hospital admission for acute exacerbations of COPD have poorly been studied.

This study was designed to study the feasibility and safety of scheduled early (two months post- discharge from acute exacerbation) home-based PR program with outpatient supervision every two weeks including exercise training and lecture series on exercise capacity and quality of life in patients with COPD. We also investigated the first use and utility of the Arabic-translated Short Form-36 (SF-36), a questionnaire designed to assess generic quality of life[7] in a brief and comprehensive manner, to assess HRQL following PR and to compare that with another specific tool, the Arabic-translated Chronic Respiratory Disease Questionnaire (CRQ).

## Methods

Design: Randomized clinical trial in a group of Arabic- speaking COPD patients.

Local institutional ethics approval was obtained before commencing the study and all enrolled patients gave written informed consent. Between July 2008 and March 2009, we recruited 39 patients admitted to the Chest Department in a tertiary hospital with moderate to severe COPD according to GOLD 2007[8] criteria for diagnosis of COPD.

Inclusion criteria were: Arabic as the first language, age over 40 years, living within local district and ability to complete the CRQ within one session.

Exclusion criteria: Patients unable to read or write, patients with locomotors problems, cognitive impairment, ischemic heart disease, aortic valve disease, cancer or lung diseases other than COPD were excluded.

The first group of patients consisted of 25 patients. In these patients we pilot tested the CRQ formats during the translation and adaptation process after stabilization of their acute illness and before their discharge. During admission period all patients received standard treatment, including nebulized bronchodilators, oxygen, oral or intravenous antibiotics, noninvasive ventilation (if required), and a one to two week course of oral prednisolone (30-40 mg daily). Patients were discharged on optimal medical treatment and received standard follow-up every two week outpatient appointments with a pulmonary specialist.

Before discharge from hospital, patients were randomly allocated to one of the two groups: Group (1) included 25 patients who underwent early pulmonary rehabilitation program as scheduled in addition to medical treatment (rehabilitation group) and group (2) included 14 COPD patients who did not undergo rehabilitation, but were kept on usual medical care for COPD as indicated (control group).

### Pulmonary rehabilitation program

This study was accomplished in four phases; planning, assessment, implementation and evaluation of COPD patients. The applied post discharge program was based on the assessment phase, review of related literature,[1,2,3–6,9,10] available resources, and patients' culture and traditions. The objectives of the model were stated. Proper teaching, learning and training aids were developed and methods of evaluation were specified. Detailed contents of the program are summarized as follow:

### Health education and lecture series

During the hospitalization period, after stabilization of the medical condition of the enrolled COPD patients, and before their discharge, enrolled patients were interviewed four times for about one hour each. The healthy lifestyle lectures included information about normal lung anatomy and physiology, disease pathology, pulmonary medications, oxygen therapy, avoiding environmental irritants, and prevention and management of respiratory infections. Information about the disease, nutrition, proper use of inhalation therapy and general preventive measures were explained. We developed a booklet to present information in a simple way using drawings. The booklet was given to the patients and used as a reminder to support teaching and practicing at home.

### Exercise training

Before discharge, and after primary assessment measures, patients were taught to perform these exercises and instructed to do them every other day at home over a period of two months. These exercises included:

1. Respiratory muscle training e.g. diaphragmatic breathing and pursed lip breathing.
2. Endurance training (aerobic training e.g. walking and cycling).
3. Strength training and isolated muscle strength training: Upper-extremity training was performed by repetitively raising and lowering a dowel from the height of the waist to the height of the shoulders (using an interval-training regimen with repetitive periods of exercise and rest as tolerated by the patient). Six to 10 upper-body and lower-body strength exercises were used based on demonstrated weakness and fatigue in each individual subject. Stretching of hamstrings, quadriceps, calves, shoulders, neck, and lower back was performed after each exercise session.

### Baseline assessment and measures of outcome

We made baseline assessments in the 24-hour period before patients were discharged from hospital and assigned to the intervention. These measurements were repeated after two months from patients' allocation and discharge. We measured exercise capacity by the six minutes walk test. We developed and used the Arabic-version of chronic respiratory disease questionnaire-self-administered standardized format (CRQ-SAS). CRQ is a well validated tool to assess patients with COPD and often used outcome measures in pulmonary rehabilitation studies, to measure disease specific health status. We measured generic health status with the short form, 36 item questionnaire for medical outcomes (SF-36). Baseline PFTs were also measured.

### A six-minute walk test

During the 6-MWD test, an index of functional capacity, subjects were asked to walk as far as they could in six minutes. As advised in the ATS statement 2002,[11] the test was performed on a continuous rectangular hospital corridor. The patient was encouraged during the test with one of three standardized phrases used by specialized nurses every minute. If the patient was receiving oxygen therapy, the nurse carried the oxygen. The test was performed twice to eliminate any potential learning effect. Walks were conducted on the same day, with at least a 30 minutes rest period between tests. The second of the two walk distances walked was measured to the nearest meter and recorded.

### Measures for quality of life

#### The Arabic-translated CRQ-SAS

The CRQ is a self-administered questionnaire developed by Guyatt[12] measuring both physical and emotional aspects of chronic respiratory disease. It is divided into the four domains of fatigue, emotional function, mastery and dyspnea. Patients are to answer each of the 20 questions on a seven-point scale expressing the degree of disability from 1 (maximum impairment) to 7 (no impairment). The questions on the dyspnea domain are individualized, in that, patients identify five important daily activities, and report their degree of dyspnea on those activities. Higher total score and sub scores on categories indicate better health related quality of life. A change in the score (calculated by dividing the overall score by the number of items) of 0.5 on the seven-point scale, reflects a clinical significant small change. A change of 1.0 reflects a moderate change and a difference of 1.5 represents a large change.[13] Use of the CRQ, authored by Drs. Gordon Guyatt and Holger Schünemann was made under license from McMaster University, Hamilton, Canada.

### Translation and instrument development

We followed a sequential forward and backward translation approach. Two translators independently translated the English self-administered CRQ (CRQ-SA) into Arabic. On agreement on first version, we then pilot tested this version on five patients to identify difficulties in understanding. In addition, we tested various possible wordings of items, answer choices and instructions if the translation team considered more than one possible version. A translator with experience in biomedical sciences but unaware of the original English CRQ performed a back translation of the Arabic CRQ formats into the source language (English). A team of McMaster University investigators compared the back translation with the English CRQ to check for conceptual discrepancies. The translation team discussed comments from these patients and finally decided on any modifications.

#### Arabic-translated medical outcomes study - 36-item short form MOS (SF-36) quality of life scale

The SF-36 incorporates 36 items and yields eight separate sub scales.[7] The validated Arabic version of the questionnaire has been used in evaluation of QOL in patients with different chronic diseases,[14–18] but never in the evaluation of Arabic- speaking COPD patients. The questionnaire include questions related to physical functioning (10), social functioning (two), role limitations due to physical problems (four), role limitations relating to emotional problems (three), mental health (five), vitality (energy/fatigue - four), pain (two), general health perceptions (five), and change in health (one). Each sub scale score ranges from 0 to 100, with 100 representing the most desirable score. The SF-36 required about 10 minutes of the patient's time and was administered during the initial patient evaluation prior to the start of pulmonary rehabilitation and at the end of 3 weeks during the final visit with the patient.

Both questionnaires on quality of life were used twice; at the beginning and end of the study period.

#### Spirometry

Spirometry was performed using computerized Sensor Medicus Corporation Machine (CAT No. 752609, SER 54065). A standard method for test performance and interpretation was used as recommended by the American Thoracic Society (ATS).[19] Forced vital capacity (FVC), forced expiratory volume in first second (FEV_1_), forced expiratory flow (FEF_25-75%_) and FEV_1_/FVC were measured. The results were then expressed as percentage of predicated normal values for each subject after adjustment for age, sex and height.

#### Blinding

Owing to the nature of the intervention, it was not possible to blind patients or assessors. The assessors were either the investigator responsible for assignment or members of the pulmonary rehabilitation team including the pulmonary specialist and the specialized nurses who were involved in the delivery of the intervention.

### Statistical analysis

Numerical values are presented as mean plus/minus (SD) unless otherwise stated. Chi square or the Fisher's exact test, if cell sizes are small, was used in the 2 × 2 data. We compared mean values of mean differences between groups for CRQ, and SF-36 scores using the Student t test. We analyzed data on an intention to treat basis. Paired t tests were used to determine if the SF-36 scores, CRQ, PFTs measures and distance walked in six minutes differed before and after rehabilitation. All tests were two-tailed unless otherwise stated, and *P* values < 0.05 were required for statistical significance.

All statistical analyses were performed using statistical software (SPSS version 11) and the on line Epi-calc 2000 for test of proportions calculations.

## Results

Baseline demographic and clinical data of all participants are presented in Table 1; both groups were comparable as regards age, residence, smoking history, duration and severity of the disease.

**Table 1:** Socio-demographic and clinical data of participated COPD patients

Item	Group 1 rehabilitation group N = 2 (%)	Group 2 usual care control group N = 14 (%)	Total N = 39 (%)	*P* value
Age (Mean±SD)	56.96 ± 11.59	56.43 ± 9.03		0.88
Residence				
Urban	11 (44.0)	2 (14.3)	13 (33.3)	0.062
Rural	14 (56.0)	12 (85.7)	26 (66.7)	
Smoking				
Non smoker	1 (4.0)	1 (7.1)	2 (5.1)	0.37
Mild (0-10 pack/year)	6 (24.0)	4 (28.6)	10 (25.6)	0.47
Moderate (10-20 pack/year)	11 (44.0)	6 (42.9)	17 (43.6)	0.39
Heavy (> 20 pack/year)	7 (28.0)	3 (21.4)	10 (25.6)	0.47
Duration of illness (years)				
<10	10 (40)	8 (57.1)	18 (46.2)	0.24
10-20	10 (40)	4 (28.6)	14 (35.9)	0.36
20-25	5 (20)	2 (14.3)	7 (17.9)	0.50
Severity of disease				
Moderate	22 (88)	10 (71.4)	32 (82)	0.19
Severe	3 (12)	4 (28.6)	7 (18)	0.19
Grades of dyspnea at time of enrolment				
II	1 (7.1)	3 (12.0)	4 (10.3)	0.47
III	11 (78.6)	22 (88.0)	33 (84.5)	0.38
IV	2 (14.3)	0 (0)	2 (5.1)	0.18
Amount of sputum				
Small	5 (35.7)	6 (24)	11 (28.2)	0.35
Moderate	5 (35.7)	8 (32)	13 (33.3)	0.45
Large	4 (28.6)	11 (44)	15 (38.5)	0.27
Cor pulmonale				
No	17 (68.0)	8 (57.1)	25 (64.1)	0.37
Compensated	7 (28.0)	5 (35.76)	12 (30.8)	0.44
De-compensated	1 (4)	1 (7.1)	2 (5.1)	0.37
Respiratory failure				
No	8 (57.1)	7 (28.0)	15 (38.5)	0.08
Yes	6 (42.9)	18 (72. 0)	24 (69.2)	
Maintenance therapy				
Oral bronchodilators				
No	3 (12.0)	0 (0)	3 (7.7)	0.23
Yes	22 (88.0)	14 (100.0)	36 (92.3)	
Inhaled bronchodilators				
No	11 (44.4)	3 (21.4)	14 (35.9)	0.14
Yes	14 (56.0)	11 (78.6)	25 (71.8)	
Oral corticosteroids				
No	1 (4.0)	0 (0)	1 (2.6)	0.38
Yes	24 (96.0)	14 (100)	38 (97.4)	

COPD = Chronic obstructive pulmonary disease

Table 2 shows baseline and two months data of SF-36 scales in the pulmonary rehabilitation group (group 1) and usual care control group (group 2). As regard the over all change in health scale; group 1 shows statistically very significant improvement between time of enrolment and after the two months PR program (*P* < 0.001), meanwhile in group 2, the mean score of the change in health tended to get lower. Concerning the four scales of the physical component; apart from general health sub scale, three of the physical components sub scales (physical function, role physical and pain) showed statistically very significant improvement in rehabilitation group at the end of the PR program *P* < 0.001) and none of the physical components' sub scales showed significant improvement by the end of the two months usual care in group 2 (*P* > 0.05). Mental component has four sub scales. Same Table shows that only the vitality and role of emotions out of the four scales of mental component showed improvement in group 1 following the PR program (*P* < 0.05, *P* < 0.001 consecutively). Despite that emotional well being and social function scores were higher after PR program, this did not reach statistically significant value (*P* > 0.05). In group 2, none of the mean mental sub-scale scores showed improvement and higher values compared to their baseline values.

**Table 2:** Baseline and two months medical outcomes study 36-items' short form MOS (SF-36) scales

SF-36 scale	Group 1 (Rehabilitation group) N = 25			Group 2 (Usual care control group) N = 14		
		
	Enroll	2 months	*P* value	Enroll	2 months	*P* value
Change in health	46.00 ± 11.81	76.00 ± 8.78	< 0.001	35.71 ± 6.16	33.53 ± 14.62	0.71
Physical component						
Physical function	30.64 ± 10.45	75.08 ± 14.31	< 0.001	25.86 ± 26.44	28.58 ± 27.51	0.79
Role physical	19.00 ± 29.12	64.00 ± 26.10	< 0.001	5.43 ± 14.58	7.14 ± 18.16	0.79
Pain	37.20 ± 6.14	61.20 ± 11.30	< 0.001	25.00 ± 9.41	28.01 ± 8.14	0.37
General health	46.00 ± 9.90	52.00 ± 9.68	0.04	36.43 ± 7.19	34.17 ± 6.12	0.38
Mental component						
Mental health (emotional well-being)	47.36 ± 5.12	48.00 ± 5.66	0.68	39.14 ± 0.07	36.15 ± 4.17	0.31
Social function	50.72 ± 15.90	54.76 ± 13.12	0.33	44.93 ± 10.44	40.39 ± 4.34	0.15
Role emotional	3.96 ± 10.94	96.00 ± 20.00	< 0.001	14.29 ± 36.31	13.27 ± 33.37	0.94
Energy/fatigue (vitality)	50.80 ± 9.65	79.60 ± 10.89	< 0.05	46.79 ± 8.23	44.78 ± 7.33	0.50

Table 3 shows baseline and two months outcome data of the two studied groups; the pulmonary rehabilitation group (group 1) and usual care control group (group 2). By the end of the study, COPD patients who underwent two months of PR program in group 1 had statistically significant increase in their achieved six minutes walk distance compared to their baseline measures (*P* < 0.05) meanwhile the mean distance achieved by patients in the usual care group 2 was even less than their baseline values, however this decrease did not reach statistically significant value. Notably, there was also statistically significant difference in the achieved distance between group 1 and group 2 by the end of the two months, where group 1 had significant increase in the achieved distance compared to group 2 (*P* < 0.01).

**Table 3:** Baseline and two months six minutes walk distance of the two studied groups of COPD patients

Outcome measure	Group 1 (Rehabilitation group) N = 25		Group 2 (Usual care control group) N = 14		Mean differences between groups (95% CI)	*P* value
			
	Enroll	2 months	Enroll	2 months		
Six minutes walk distance in meters						
Mean ± SD	88.79 ± 19.14	141.71 ± 23.11	83.79 ± 15.9	68.56 ± 32.11	58.15 ± 11.23	< 0.001#
Chronic respiratory disease questionnaire$						
Dyspnea (range 5-35)	11.8 ± 5.0	19.6 ± 5.2*	12.4 ± 4.4	13.5 ± 4.3	5.5 (3.0-9.0)	0.003^
Fatigue (range 4-28)	9.8 ± 2.8	17.4 ± 5.4*	11.6 ± 6.1	13.2 ± 5.1	5.3 (1.9-9.8)	0.004^
Emotion (range 7-49)	22.1 ± 5.8	33.5 ± 7.2*	27.0 ± 12.6	29.7 ± 11.4	8.7 (2.5-15.0)	0.008^
Short form 36 (range 0-100)$						
Physical component	30.6 ± 14.2	56.3 ± 24.0*	40.6 ± 21.9	47.2 ± 24.2	20.1 (3.3-36.8)	0.02§
Mental component	26.3 ± 14.6	39.0 ± 20.0*	30.4 ± 19.9	32.4 ± 22.2	10.6 (-0.3-21.6)	0.047§
Pulmonary function tests (spirometry)						
FVC (L/min)	1.37 ± 0.50	1.42 ± 0.59	1.09 ± 0.41	0.98 ± 0.20	0.44 (0.11-0.77)	0.01^
FVC (% pred)	36.24 ± 14.17	40.4 ± 16.16	29.0 ± 10.91	26.57 ± 7.13	13.83 (2.5-14.9)	0.00^
FEV1 (L/min)	0.80 ± 0.35	0.83 ± 0.52	0.62 ± 0.18	0.64 ± 0.20	0.19 (-0.1-0.48)	0.2§§
FEV1 (%pred)	29.44 ± 13.14	29.92 ± 20.21	23.21 ± 7.70	23.14 ± 7.56	6.78 (-5.01-18.57)	0.25§§

#Data expressed as mean score (SD);

$ Increased score denotes improvement; *P* < 0.05 between baseline and two months in rehabilitation group using paired *t* test;

# *P* < 0.001 between group 1 and group 2 after two months of enrolment using unpaired *t* tests;

^ *P* < 0.01 between group 1 and 2 after two months of enrolment using unpaired *t* tests;

* *P* < 0.05 enroll vs 2 months in group two using paired *t* tests;

§ *P* < 0.05 between group 1 and 2 after twomonths of enrolment using unpaired *t* tests;

§§ *P* > 0.05 between group1 and 2 after two months of enrolment using unpaired *t* tests, COPD = Chronic obstructive pulmonary disease

Chronic respiratory disease questionnaire domain scores of the two studied groups are shown in the same table [Table 3]. Increased score denotes improvement. Minimal clinically important difference is 2.5 (dyspnea domain), two (fatigue domain), 3.5 (emotion domain) and two (mastery domain).[20] There were statistically and clinically significant increase in the four CRQ domains mean scores in group 1 by the end the two- month PR program (*P* < 0.05). Also, statistically significant differences were noticed between group 1 and 2 by the end of the study (*P* < 0.01). The *P* values between the two groups were as follows: In mastery (*P* < 0.001), dyspnea (*P* = 0.003), fatigue (*P* = 0.004) and lastly emotion (*P* = 0.008).

Table 3 also shows baseline and two months SF-36 components mean scores of the two studied groups. There was statistically significant increase in the mean score of both SF-36 physical and mental components in group 1 at the end of the two-month PR program compared to their baseline mean scores values (*P* < 0.05). Also, there were significant differences in the mean scores between group 1 and 2 by the end of the study (*P* < 0.05). This difference was more marked in the physical component (*P* = 0.02) than in the mental component (*P* = 0.047).

Baseline and two-month PFT data of the two studied groups are shown in Table 3. By the end of the two months, there were no significant differences between mean values of spirometeric data variables between group 1 and 2 as regards FEV_1_ and FEV_1_% (*P* > 0.05). However, FVC and FVC percentage were significantly higher in rehabilitation group compared to control group (*P* < 0.05). Also, despite that FEV_1_, FEV_1_ percentage of predicted, FVC and FVC percentage of predicted improved slightly in group 1 following the two months rehabilitation program, this improvement was not statistically significant (*P* = 0.79,).

## Discussion

Hospital admission for acute exacerbation of COPD is an enormous financial burden to health service. The current evidence shows that PR provides significant benefits to patients.[1–6,9,21]

The baseline measurements of age and disease severity of this sample of COPD patients included in this study were similar to those of other studies assessing PR. The baseline scores for the CRQ and the SF-36 were also similar to those measured in individuals with COPD in previous studies.[1,2,4–6,9,10,20]

This study proves that early PR in the recovery period after hospital discharge following an admission for an acute exacerbation of COPD leads to significant improvement in functional capacity and QOL in those patients. It adds that either the commonly used specific (CRQ) or generic (SF-36) quality of life tools could be used as an outcome measure for quality of life; hence, broadening of the comparison between patients with various chronic diseases would be feasible.

### Comparison with other studies

Excellent evidence supports the benefits of PR in stable patients with COPD.[1,3–6,20,22] Nevertheless, it is not yet widely utilized in many developing countries.[23] This study examines the effects of this intervention in patients during the early recovery period after a hospital admission for an acute exacerbation. Despite optimal medical treatment during hospital admission, patients at discharge take considerable time to recover to baseline levels of physical functioning and health status. Previous studies have shown that up to 25% of patients after acute exacerbations do not fully recover to baseline peak flow at three months[24] and that the recovery period in health status is long even in patients who do not have further exacerbations.[25] Our data indicates that patients can safely participate in a supervised, home-based PR program shortly after an exacerbation and that such a program speeds up recovery from the debilitating effects of a hospital admission. Furthermore, the magnitude of the effects of early PR on exercise capacity and health status are considerably great.

A few studies investigated exercise training after an acute exacerbation of COPD. Man *et al*. studied the effects of three- month PR program on an outpatient basis and the likelihood of financial benefit to the health service.[2] Early PR, compared with usual care, led to significant improvement in median incremental shuttle walk distance, St. George's Respiratory Questionnaire, CRQ the mental component of the SF-36 score. Also, Behnke *et al*. looked at the effects of an initial, 10-day, inpatient training program, followed by six months of supervised home training, compared with usual care, in patients admitted for an acute exacerbation of COPD.[26] They showed improvement in six-minute walking distance and sum scores on the questionnaire on chronic respiratory disease at three months and six months after training compared with control. However, such a program would not be viable in terms of manpower or finance. In contrast, especially in a low income country, two months scheduled home-based PR program with outpatient supervision every two weeks is a more realistic option to save healthcare resources. Previous data support the cost effectiveness of home-based PR program and the likelihood of improvement in the functional status and quality of life that would in turn have positive impact on health service.[5,23]

### Six-minute walk test

In patients with chronic lung disease, the minimal clinically important improvement in 6MWD has been reported[27] to be 54 m. A meta analysis[28] of PR participants has shown a similar minimal clinically relevant increase in 6MW distance 55.7 m.

We found a statistically significant improvement in 6-MWD after rehabilitation, which has also been demonstrated in previous studies,[21,28] signifying improvement in patients' functional status. By the end of two months from enrolment, the rehabilitation group showed statistically significant improvement in 6-MWD compared with their baseline value (52.62 ± 11.2). The mean difference in walk distance between the rehabilitation group compared to usual care control group was (58.15 ± 11.23).

Several factors may influence the 6MWD in healthy individuals and in COPD patients. Patient's height, spirometric parameters and diffusion capacity correlates with the achieved 6-min walk distance.[29] Also, body weight, age, mental health, and co morbidities can affect the test results in elderly individuals.[30] The sensation of breathlessness (dyspnea)[31,32] and poor nutritional state[33] are manifestations of COPD that can also reduce 6MWD. Muscle strength in the lower limbs has previously been shown to be an important factor in determining the 6MWD.[34,35] In addition, when the primary respiratory muscles are dysfunctional or cannot meet the ventilatory demand, muscles whose principal function is to maintain posture may assume an accessory muscle function.[36,37] The trapezius, latissimus dorsi, pectoralis major, and serratus anterior can all function as inspiratory muscles.[36,37]

There was minor clinically significant improvement in the six- minute walk distance following PR, yet, the average walk distance of the cohort is still considered very short. This apparently short distance might be attributed to the facts mentioned above and the facts that enrolled patients were at advanced stage of their illness, many of them were in grade III or IV, and they were just coming out of an acute exacerbation of their chronic illness. Also, at time of enrolment, 69.2% had respiratory failure and 35.9% had decompensate cor pulmonale which might be additional two factors contributing to the short distance achieved by patients with chronic illness.[38–40] Also, the end point of assessment of the patients was by the end of two months from enrolment, and possibly this outcome might has been differed if we applied the program for longer period or if the patients were assessed at multiple/longer terms. Moreover, as we mentioned above, we had no control over the level of intensity of the exercise practiced at home, where some of the patients practiced lower intensity exercise regimens that might had an effect on the overall mean six-minute walk distance of others who practiced in higher intensity. However, similar minimal clinically relevant increase in 6MW distance has been demonstrated by other investigators.[28]

### Health related quality of life

By the end of the study, the differences between group 1 that underwent the two-month PR program and group 2 were statistically and clinically significant for all CRQ domains, SF 36 physical and mental components.

### CRQ

It is necessary that change after an intervention be clinically important as well as statistically significant. Jaeschke *et al*. defined the minimally clinically important difference as “the smallest difference in score in the domain of interest which patients perceive as beneficial.”[38]

This is the first study to use the Arabic-translated CRQ-SA. The CRQ demonstrated changes that were calculated to be clinically important in both the physical function and emotional function components. The improvement noted in QOL, as measured by the physical function categories of the CRQ, was consistent with the changes found in previous studies of pulmonary rehabilitation using the CRQ.[5,12,39] In addition to improvement in physical function, the CRQ recorded changes in emotional function that were also consistent with those from previous studies.[12,39]

The original CRQ does not address the ability of an individual to perform activities that are routinely performed in daily life, such as walking, climbing stairs, dressing or bending. This may be a limitation of the earlier questionnaire. However, the CRQ- SA questionnaire uses standardized dyspnea domains. The standardized dyspnea domains produce higher cross-sectional correlations than the individualized dyspnea domains. This finding is important because it indicates that the standardized CRQ dyspnea domain allows for better discrimination between different degrees of COPD severity. Also, the SF-36 addressed physical ability. Thus, we believe that the use of both questionnaires in our study broadened the content validity of the quantitative aspect of the QOL assessment. This concept actually is in agreement with other authors conclusions from previous studies.[39,40]

### SF-36

Patients in group 1 showed both statistically and clinically significant improvements in SF-36 physical and mental components summary scores from PR entry to two months of PR participation.

Three of the physical component sub scales (physical function, role physical on limitation of activities and pain) showed statistically significant improvement in rehabilitation group; meanwhile, only the vitality and role of emotions out of the four scales of mental component showed improvement in the mental function component. The overall change in health was significantly improved in rehabilitation group (*P* < 0.001).

Some investigators noted that PR participants perceive greater impairment in the physical aspects of their health rather than in the mental aspects, and that they show greater physical improvements rather than psychosocial improvements following both short-term and long-term PR.[41] Our findings are similar to those of Benzo *et al*. - SF-36 physical and mental component summary scores to improve in 22 patients with COPD following six weeks of supervised outpatient PR. Our patients had baseline mental component summary scores nearly similar to those of Benzo *et al.* with mean scores in the 30s.[41] Nevertheless, we observed mental improvement to a smaller extent after two months of participation in PR program. These small improvements may have been because psychosocial improvements may take longer to be appreciated than physical improvements in PR participants. Also, on comparing the two questionnaires, the key difference between them was that the CRQ asked the individual to measure fear, panic and anxiety when short of breath, as well as the individual's sense of control and confidence over COPD. In contrast, the SF-36 had no questions that related to dyspnea, panic or gaining control. It is likely that the questionnaire could not detect these changes, which may account for the relative lack of responsiveness of the mental health summary component of the SF-36.

### Spirometry

Despite that FEV_1_, FEV_1_% of predicted, FVC and FVC% of predicted improved slightly in group 1 following the two-month rehabilitation program, this improvement was significant for FVC and FVC% predicted but not statistically significant for FEV_1_, FEV_1_% of predicted. This agrees in part with the previous study of Safwat *et al*. who reported that there was significant change in FVC, FVC%, FEV_1_ and FEV_1_% after the rehabilitation program related that to the anabolic steroid which was added to exercise rehabilitation program.[10]

### Limitations

First, this study was confined to the immediate benefits of PR program applied for COPD patients following recent acute exacerbation. It did not measure the effect of this program on frequency of exacerbations, rate of re admissions to the hospital or the long term effect.

Also, we had no control over the level of intensity of the exercise practiced at home. While some patients could have incorporated higher intensity, others may have employed lower intensity exercise regimens. However, studies have shown that both low-intensity and high-intensity exercise training improves QOL and physical performance parameters in patients in PR programs.[42] Thus, looking at the variation in exercise intensity practiced by the patients may not have played a significant role in this respect.

Lastly, the cohort of this study was small because only patients literate enough to complete the questionnaires by themselves were included. Despite that this is the first study to use the Arabic translation version of CRQ-SA and SF-36 in COPD patients, and because of the limited number of patients included in this study, it would be worthwhile if those two Arabic assessment tools be validated in another study with higher number of patient, and to add other interviewer- administered tools like CRQ-IA to be able to assess any patients with chronic respiratory diseases, regardless their levels of educations in any future Arabic studies.

## Conclusions

Despite medical optimization during hospital admission for acute exacerbations of COPD, early PR after discharge from hospital leads to additional notable improvements in exercise capacity and health status at two months compared with usual care. The 6MWD is a simple, inexpensive, reliable and safe test to assess physical and functional capabilities among COPD patients. HRQL can be measured in patients with COPD either by disease-specific tools that have been specifically designed for use in patients with respiratory system disorders or by generic HRQL tools that can be used across populations with a variety of medical conditions allowing comparison of the results of pulmonary rehabilitation to therapeutic interventions in patients with other medical disorders.

Larger randomized studies are required to determine translation of benefits of early pulmonary rehabilitation into improved health economics. Other unanswered questions include long term effects of early PR, and the optimal structure, location, and duration of PR program in low income countries.

## Abbreviations

Chronic Obstructive Pulmonary Disease = COPD; Pulmonary Rehabilitation = PR; 6 MWD = six-minute walk distance; CRQ = Chronic Respiratory Disease Questionnaire; CRQ-SAS = chronic respiratory disease questionnaire-self-administered standardized format; QOL = quality of life; HRQL = health-related quality of life; SF-36 = Short form-36 = MOS (SF- 36) = Medical Outcomes Study 36-item short form; PFTs = pulmonary function tests; ABGs = arterial blood gases
